# Acute Isolated Hyperbilirubinemia as a Presentation of Alcoholic Liver Disease: A Case Report and Literature Review

**DOI:** 10.1155/2018/9403934

**Published:** 2018-02-12

**Authors:** Muhammad Rajib Hossain, Ramesh Kumar Pandey, Mohammad Faridul Islam, Praveen Datar, Vijay Gayam, Pradeep Puri, Thwin Malar

**Affiliations:** Department of Medicine, Interfaith Medical Center, Brooklyn, NY, USA

## Abstract

Isolated hyperbilirubinemia as a manifestation of alcoholic liver disease without significant liver abnormalities is seen very rarely. We report such a case where a patient with chronic alcoholism presented to the ER with acute jaundice with bilirubin of 24.8 mg/dl, predominantly conjugated in nature along with mild elevation of AST (76 IU/L). There were no other abnormalities of the liver function. The patient underwent extensive laboratory and imaging tests that excluded extrahepatic cholestasis, viral and autoimmune hepatitis, ischemic hepatitis, and so forth. Liver biopsy excluded hemochromatosis, dysplasia, or malignancy and other differentials. Bilirubin gradually trended down to 7.3 mg/dl when alcohol consumption was stopped.

## 1. Introduction

Excessive alcohol consumption is a common cause of liver dysfunction and can lead to significant morbidity and mortality from end-stage liver failure [1]. Although the consumption of >30 g/day of alcohol (defined as at-risk drinking) has point prevalence of cirrhosis 1%, alcohol was responsible for 48% of all deaths from cirrhosis as per the National Institutes of Health estimation in 2009 [2]. Alcoholic liver disease has diverse manifestations ranging from alcoholic fatty liver disease (with or without steatohepatitis) to alcoholic hepatitis and cirrhosis. Most of the patients are typically asymptomatic at the initial stage, and as disease advances they can present with symptoms including jaundice, other signs of peripheral stigmata of liver disease, or signs of hepatic decompensation depending on severity [3]. The case we are presenting here had an unusual presentation of only jaundice without significant abnormalities in other liver functions. Due to the rare presentation, the patient underwent extensive tests to rule out other differentials.

## 2. Case Report

A 63-year-old African American male with a history of chronic alcoholism presented to the ER after noticing a yellowish discoloration of the skin, dark urine, and worsening nausea, as well as anorexia for the last 7 days. He denied associated abdominal pain or abdominal distention.

He did not have associated abdominal pain, abdominal distention, pedal edema, passage of black tarry stool, bloody vomitus, fever, or rash. He also experienced generalized itchiness. He never had such jaundice before nor did his family members have such jaundice or any known liver diseases. He was not taking any medication. He has been drinking alcohol for the last 45 years, about one and a half pint of vodka daily. Physical exam was only notable for deep icteric sclera, but abdomen exam was unremarkable and there were no peripheral stigmata of liver disease and his BMI was 23. Laboratory findings were significant for total bilirubin of 24.8 mg/dl with direct bilirubin of 18.8 mg/dl, AST of 76 IU/L (Figure 2), platelets of 28 K, and albumin of 2.7 gm/dl, and the rest of the laboratory findings were within the normal limits, including ALT, ALP (Figure 2), GGT, MCV, INR, and PT. His Hb was 9.9 gm/dl with a baseline of 10; MCV was 90 Fl; peripheral smear did not show schistocytes.

**Figure 1:**
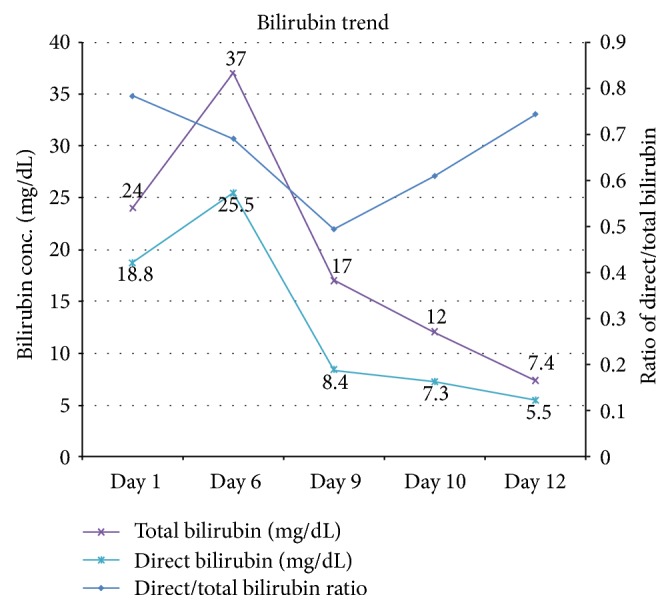
Trend of bilirubin (total and direct) during the course of hospitalization.

**Figure 2:**
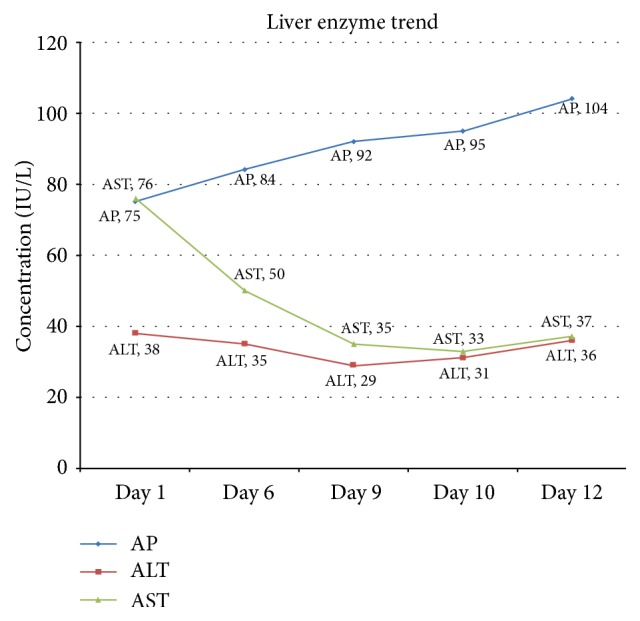
Trend of AST/ALT/ALP during the course of hospitalization.

**Figure 3:**
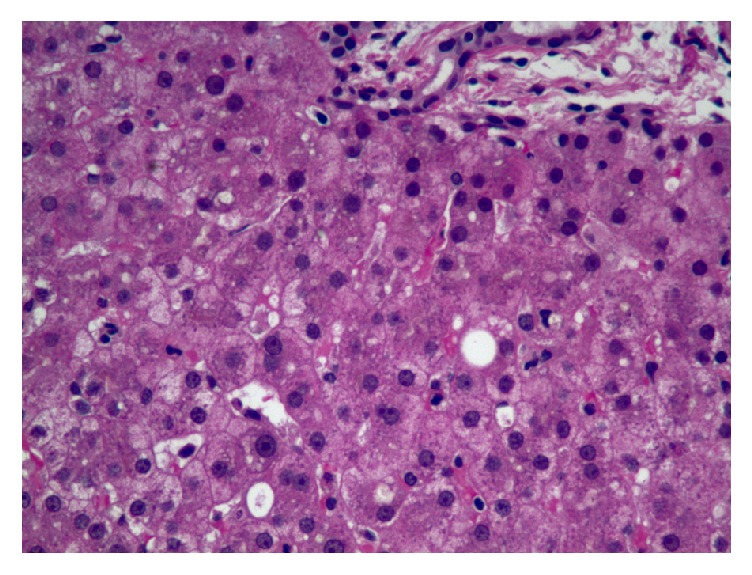
Liver biopsy showed focal steatosis, focal nonspecific chronic inflammation, and a nonspecific increase in the lipochrome pigment in hepatocytes.

On admission, the differentials were extrahepatic cholestasis versus intrahepatic cholestasis (alcoholic liver disease, viral hepatitis). Abdominal sonogram showed a small right hepatic cyst (1.2 cm) without evidence of cirrhosis or extrahepatic duct obstruction. Abdominal imaging including CT and MRI of the liver with MRCP was also unremarkable. Hepatitis viral panel was negative for hepatitis A, B, C, and E viruses. Other viral panels—CMV, EBV, parvovirus, and AMA (anti-smooth muscle antibody)—and sickle cell screen were negative. ERCP and endoscopic ultrasound (EUS) were not performed due to the low diagnostic pretest probability, considering cost, and were reserved as the last tool. Liver biopsy (Figure 3) excluded hemochromatosis, dysplasia, or malignancy. It (Figure 3) reported focal steatosis, focal nonspecific chronic inflammation, and portal fibrosis and a nonspecific increase in the lipochrome pigment in hepatocytes. Bilirubin gradually trended down to 7.3 mg/dl after days (Figure 1, trend of bilirubin). The patient was counseled on alcohol abuse and discharged with instructions and recommendations to follow up in a GI clinic. However, the patient did not comply with the followup schedule.

## 3. Discussion

Alcoholic liver disease has diverse presentation ranging from alcoholic fatty liver disease (with or without steatohepatitis) to alcoholic hepatitis and cirrhosis. Clinical jaundice or histological features of intrahepatic cholestasis can be seen in different stages of alcoholic liver disease [3]. However, profound jaundice or cholestasis without abnormalities of liver functions especially without a significant elevation of AST and features of cirrhosis is rarely reported as seen in our patient. Based on our search for alcohol related cholestasis using multiple search engines including Google Scholar, we have found a single abstract presented by Dr. Edwards at the American College of Physicians' conference on May 1, 1968. In his case series, he presented relatively benign and excellent recovery among patients with isolated cholestasis in comparison to those with potentially life-threatening alcoholic hepatitis [4].

Bile formation and secretion is a complex process starting from the bone marrow to the liver. Bilirubin is a product of heme degradation which is conjugated in the liver and is then finally secreted in the bile duct as bile salts. The transport of bile from hepatocytes to biliary canaliculi is via bile salt dependent and independent pathways [5]. Based on the rat model studies mainly, the bile salt independent pathway needs chemical and electrical driving forces generated by the Na-K ATPase in the basolateral membrane [6]. Na-K ATPase is inhibited by alcohol. Based on some studies, alcohol also impairs transcytosis within the hepatocytes [7, 8]. The exact pathology behind alcohol induced intrahepatic cholestasis is not well established, but experts identified the possible causes as interference with basolateral uptake and intracellular transport of bile acids or compression of the intrahepatic biliary tree [9]. Further studies are needed to understand the pathology behind alcohol induced intrahepatic cholestasis.

In most cases, alcohol induced liver disease can be diagnosed easily with a reliable history, standard liver function tests, and imaging tests [10, 11]. In reference to biopsy confirmed alcoholic liver disease patients, more than 90% of them have AST/ALT ratio of more than 2 in laboratory testing [12]. Biopsy rarely indicates to diagnosing alcoholic liver disease and it has been found that there are considerable histologic similarities between the alcoholic and nonalcoholic patients and they are often indistinguishable based on histology alone [13]. Gamma-glutamyl transpeptidase (GGT) is a very sensitive test though nonspecific for alcohol related liver disease which is elevated in almost all patients with alcoholic liver disease [14]. Having this test normal added more to the diagnostic dilemma in our case.

Followup in such cases is important to see how fast bilirubin normalizes prognosis and identifies complication and any other factors retrospectively relating to the case. One of the drawbacks in our case report is that we do not have followup information as the patient did not come to the clinic for followup. This also emphasized the fact that loss to followup could be a problem in managing patients with alcohol abuse and those who need more intense focus, counseling, and strategy to encourage them to comply with the followup plan to prevent future complications from liver disease.

There have been reports of cholestasis with acute alcoholic liver disease, but isolated elevation of bilirubin might be the only manifestation of the alcoholic liver disease as in our case [15]. Most of the time, history and physical examination guide the diagnosis of alcohol related liver disease. However, in some cases like ours, diagnosis can be challenging due to the very atypical presentation, and such cases may need more invasive studies such as liver biopsy or ERCP to diagnose. Denial of alcohol intake can also complicate the diagnostic dilemma. Painless jaundice, mostly being a harbinger of cancer in the hepatobiliary tract, can sometimes be due to alcohol ingestion as in our case.
